# Comprehensive Assessments in Bonding Energy of Plasma Assisted Si-SiO_2_ Direct Wafer Bonding after Low Temperature Rapid Thermal Annealing

**DOI:** 10.3390/mi13111856

**Published:** 2022-10-29

**Authors:** Youngseok Lee, Yebin You, Chulhee Cho, Sijun Kim, Jangjae Lee, Minyoung Kim, Hanglim Lee, Youngjun You, Kyungman Kim, ShinJae You

**Affiliations:** 1 Applied Physics Lab for Plasma Engineering (APPLE), Department of Physics, Chungnam National University, Daejeon 34134, Korea; 2 Advanced Components Research Lab, SEMES, Hwaseong-si 18383, Korea; 3 TP Advanced Equipment Group, SEMES, Cheonan-si 31040, Korea; 4 Institute of Quantum Systems (IQS), Department of Physics, Chungnam National University, Daejeon 34134, Korea

**Keywords:** wafer bonding, silicon, silicon dioxide, plasma treatment, plasma-surface interaction

## Abstract

Direct wafer bonding is one of the most attractive techniques for next-generation semiconductor devices, and plasma has been playing an indispensable role in the wider adoption of the wafer bonding technique by lowering its process temperature. Although numerous studies on plasma-assisted direct wafer bonding have been reported, there is still a lack of deep investigations focusing on the plasma itself. Other than the plasma surface treatment, the wafer bonding process includes multiple steps such as surface cleaning and annealing that require comprehensive studies to maximize the bonding strengths. In this work, we evaluate the various process steps of Si-SiO_2_ wafer bonding through case-by-case experimental studies, covering factors including the plasma conditions for surface treatment and secondary factors such as the time intervals between some process steps. The results show that plasma treatment with increasing input power has a trade-off between bonding strengths and interfacial voids, requiring the optimization of the plasma conditions. It is also noticeable that the effects of plasma treatment on wafer bonding can be improved when the plasma-treated wafers are stored in ambient atmosphere before the subsequent process step, which may suggest that wafer exposure to air during the bonding process is advantageous compared to processing entirely in vacuum. The results are expected to allow plasma-assisted direct wafer bonding technology to play a bigger role in the packaging process of semiconductor device manufacturing.

## 1. Introduction

Recently, semiconductor packaging technology has attracted great interest for the manufacturing of state-of-the-art semiconductor devices to meet market demands including faster operation speeds and higher functional densities of semiconductor chips [1,2]. The advanced packaging technology, including fan-out wafer level packaging and through-silicon via, allows for three-dimensional (3D) device integration that leads to improved device performance and miniaturized system electronics [3,4,5]. Direct wafer bonding is one of the most essential techniques to build stack-up structures of manufactured semiconductor chips in the packaging process [6,7,8]. Both homogeneous and heterogeneous integrations between diverse semiconductor materials and Si substrates can be achieved via the direct wafer bonding technique, enabling numerous applications such as silicon-on-insulator fabrication and silicon-based microelectromechanical device manufacturing to be more widely employed [9,10,11].

As its name denotes, direct wafer bonding refers to a technique that creates a wafer pair in which the surfaces of two wafers adhere to each other without any additional intermediate layers, which is an advantage in terms of the throughput and accuracy of 3D integration [7]. There have been numerous reports on direct wafer bonding considering target materials, procedures, etc., since the early 1960s when high-temperature (>800 °C) annealing was often employed to achieve sufficient bonding strengths [12,13]. Nowadays, plasma-assisted wafer bonding has emerged as a new trend since plasma treatment allows the wafer bonding process to be conducted without high-temperature annealing that involves several serious disadvantages such as device reliability and manufacturing yield [12,13,14,15]. Plasma-assisted wafer bonding with low temperature (~300 °C) annealing yields bonding strengths equivalent to those obtained in the conventional way of high temperature annealing [16,17].

Despite this remarkable advantage though, few previous reports have focused on the plasma itself in plasma-assisted wafer bonding. Several studies have shown experimental evidence of the effects of plasma treatment on bonding strengths, but most experiments were conducted with insufficient details of the plasma characteristics [14,15,18,19,20]. Since the physical and chemical properties of plasmas are significantly dependent on the environment, it is important to optimize not only the processing plasma in plasma-assisted wafer bonding to maximize the effects of the plasma treatment but also the other process steps including surface cleaning and annealing.

In this work, we conduct comprehensive assessments of the wafer bonding process where multiple individual steps should be carefully controlled. The investigation is especially focused on the plasma treatment step, which is shown in this work to have the greatest effect on the bonding strength. Detailed descriptions of the investigation are as follows. In Section 2, the experimental setup of the plasma treatment chamber and RTA are described, and in Section 3, the assessment results of each step of the wafer bonding process are presented. Concluding remarks follow in Section 4. 

## 2. Experimental

### 2.1. Plasma Treatment

Plasma treatment is conducted in a capacitively coupled plasma chamber that has a cylindrical geometry with a diameter of 340 mm and a height of 145 mm. With N_2_ as a reference gas, Ar and O_2_ are also used for investigation of the effects of different plasmas on bonding strengths, as discussed in Section 3.2.1. Wafers are loaded on the bottom electrode with a height of 65 mm and a radius of 335 mm where radiofrequency (RF) powers are applied, while the top grounded electrode, which acts as a showerhead as well, faces the bottom electrode at a distance of 50 mm. Further details of the plasma system are described in our previous reports [21,22,23]. Prior to plasma ignition, the chamber is evacuated by a turbomolecular pump for 5 min, resulting in a pressure on the order of 10^−5^ Torr. Mass flow controllers inject processing gases into the chamber at fixed flow rates, and a throttle valve maintains a constant chamber pressure during the plasma treatment. The door-to-door time for the plasma treatment of one wafer from wafer loading to chamber venting is approximately 15 min.

### 2.2. DIW Cleaning

Plasma-treated wafers are transferred to a clean room at ambient atmosphere at 23 °C with RH 43%. To remove contaminants such as dust on the wafer surfaces, DIW cleaning is conducted using a spinning vacuum chuck with a diameter of 300 mm on which wafers are strongly held by a diaphragm pump. DIW is applied on rotating wafers through the nozzle of a wash bottle, followed by a spin-drying step to remove the DIW on the wafer surfaces.

### 2.3. Prebonding

Cleaned and dried wafers are transferred to an apparatus, named the prebonder, to create a ‘prebond’ between the surfaces of two plasma-treated wafers prior to the annealing process. A Si wafer is first put on the stage of the prebonder with its plasma-treated surface facing up, and then a SiO_2_ wafer that is flipped so that its plasma-treated surface faces down is put onto the Si wafer, producing a wafer pair where two plasma-treated surfaces are mated to each other. The contact between the mating surfaces is strengthened by applying a weak local pressure to the center of the wafer pair by finger for tens of seconds and then to the entire area for one minute for stronger van der Waals adhesion before annealing.

### 2.4. Annealing

Wafer pairs that have passed through plasma treatment, DIW cleaning, and prebonding are annealed in the RTA, which has a square geometry with an inner width and length of 318 mm. Wafer pairs are loaded into the chamber with a loadlock that is made of a quartz ring with an inner and outer diameter of 290 and 305 mm, respectively. Halogen lamps heat the chamber by infrared radiation through a 16 mm thick quartz plate, increasing the temperature up to near 400 °C, which is monitored with a K-type thermocouple gauge laterally distant from the edge of the quartz holder by 5 mm. The annealing time was determined by comparing the bonding strengths from different annealing periods, as mentioned in Section 3.5. After wafer pairs turn into bonded wafers via annealing, they are taken out of the RTA to ambient atmosphere when the RTA temperature reaches under 80 °C after cooling down for 20 min. The wafer bonding process is completed with annealing as the final step.

### 2.5. DCB Evaluation

The DCB method suggested by Maszara et al. is employed to evaluate the bonding strengths of the bonded wafers [24]. Wafers are loaded on the vacuum stage of the DCB equipment to prevent them from being pushed backward during the evaluations. A blade with a thickness of 5 mm, which is controlled by an electrical motor, pierces cracks in the interfaces of the bonded wafers, penetrating 5 mm in depth. The cracks in the bonded interface from the blade insertion appear in 1200 nm infrared (IR) images as dark segments in the wafer circle. The maximum width of the segments is obtained from the IR images in the unit of mm, followed by conversion to bonding strength in units of J/m^2^ via the Maszara formula, which is given by [25]

(1) $$\gamma =3Et^{3}\;y^{3}/\left(8L^{4}\right)$$

where *γ* is the specific surface energy in ergs/cm^2^, *2y* and *L* are the crack separation and length, respectively, *t* is the thickness of the wafer, and *E* is the modulus of elasticity in Gpa. Note that the surface energy is referred to as the bonding strength in this paper.

### 2.6. Characterization

Optical emission spectroscopy (OES) is employed to characterize the vacuum condition prior to plasma treatment. An optical fiber is mounted laterally on one of the chamber ports and monitors the inside of the chamber through a quartz window. The detected optical signals are transferred through the optical fiber to a spectrometer (QEPRO-FL, Ocean Insight, Orlando, FL, USA). The electron density is measured with a cutoff probe, of which reliability has been proven in several reports [26,27,28,29,30,31]. Details of the probe geometry are also introduced in a previous report [32]. One cutoff probe is inserted into the vacuum chamber through a feedthrough of another chamber port and is connected to a network analyzer (S33601B, Saluki Technology, Taipei, Taiwan). The self-bias voltage is obtained with a high-voltage probe (P5100, Tektronix, Beaverton, OR, USA) that measures the voltage in the middle of the power feeding copper rod between the impedance matcher and the powered electrode.

## 3. Results and Discussion

**Figure 1:**
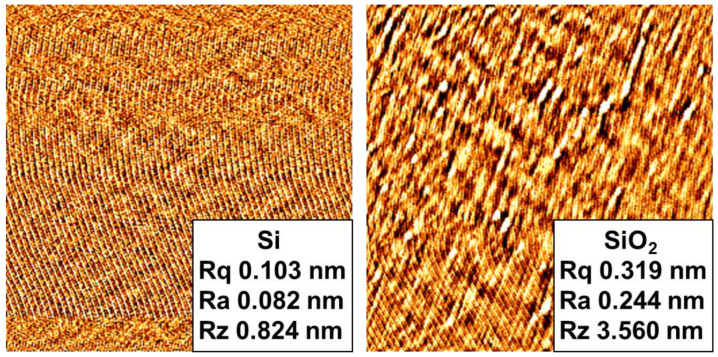
Surface roughness of Si and SiO_2_ wafers before plasma treatment.

**Figure 2:**
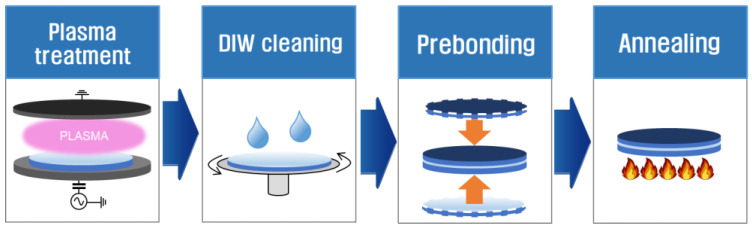
Sequence of the wafer bonding process.

The sequence of the bonding process employed in this work is illustrated in Figure 1. We use Si and SiO_2_ wafers with a diameter of 300 mm, which are among the most widely used wafers in semiconductor device manufacturing. One representative surface analysis result on Si and SiO_2_ surfaces is presented in Figure 1. We assume that the status of the Si and SiO_2_ wafers at each bonding trial is almost identical. Their roughness is measured by atomic force microscopy (XE-120, Park Systems, Suwon, Korea). Starting with plasma treatment on the wafer surfaces, deionized water (DIW) cleaning follows to remove contaminants such as dust from the wafer surfaces before putting them together. The prebonding step in Figure 2 refers to a process where two plasma-treated and DIW cleaned wafers are completely overlapped with the to-be-bonded surfaces mated to each other, which are kept in contact by attractive van der Waals interactions at the interface. The prebonded wafer pairs are then transferred to rapid thermal annealing equipment (RTA) and annealed to form siloxane (Si-O-Si) bonds at the interface between the wafers, creating a newly fused interlayer [33]. The resulting bonding strengths are evaluated with a double cantilever beam (DCB) method [25]. It should be noted that the above process steps proceed in ambient atmosphere except the plasma surface treatment and annealing steps.

**Figure 3:**
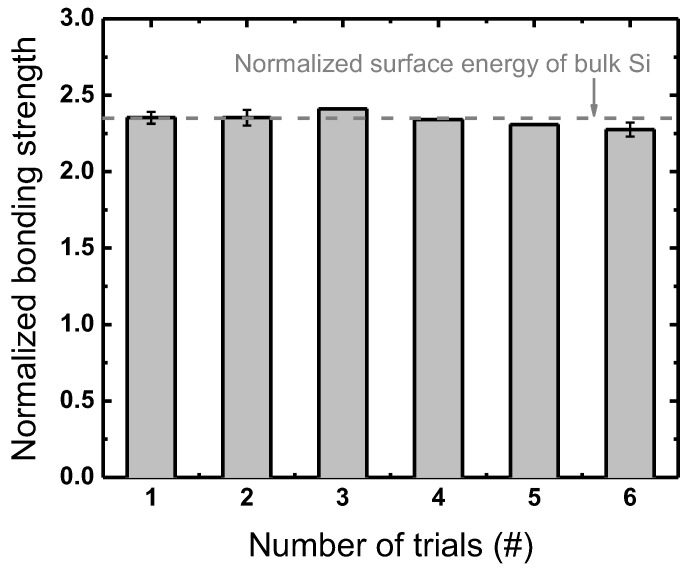
Bonding strengths obtained through an identical process under the same processing conditions normalized to the bonding strength with no plasma treatment.

Prior to process assessment, reference conditions throughout the wafer bonding process that provide a good reproducibility from repeated operations should be determined. Figure 3 plots the results of an examination for the reference setup conducted under the conditions listed in Table 1. Note that the bonding strengths, also referred to as surface energy or bond energy, are typically given in the physical unit of J/m^2^, but in this work they are normalized to the bonding strength with no plasma treatment. The error bars in the figures indicate the standard deviation of the bonding strengths measured at the cardinal points on each wafer pair at every trial. Reproducibility is assessed with a statistical approach to the DCB results from six trials of wafer bonding all at the reference condition, and the results show an averaged normalized bonding strength of 2.34 with a standard deviation of 0.04 (approximately 2%), which is considered to be acceptable. Thus, the conditions listed in Table 1 are set as the reference.

In this section, we provide the results of our process assessment. Following the order of the wafer bonding process illustrated in Figure 1, the results of the step before plasma treatment are given in Section 3.1, followed by a discussion on the results of the plasma treatment step in Section 3.2. Section 3.3 and Section 3.4 describe the results of the steps between the plasma treatment and prebonding and between prebonding and annealing, respectively. Finally, the results of the annealing step are presented in Section 3.5.

### 3.1. Before the Plasma Treatment Step

#### 3.1.1. Surface Rinsing

**Figure 4:**
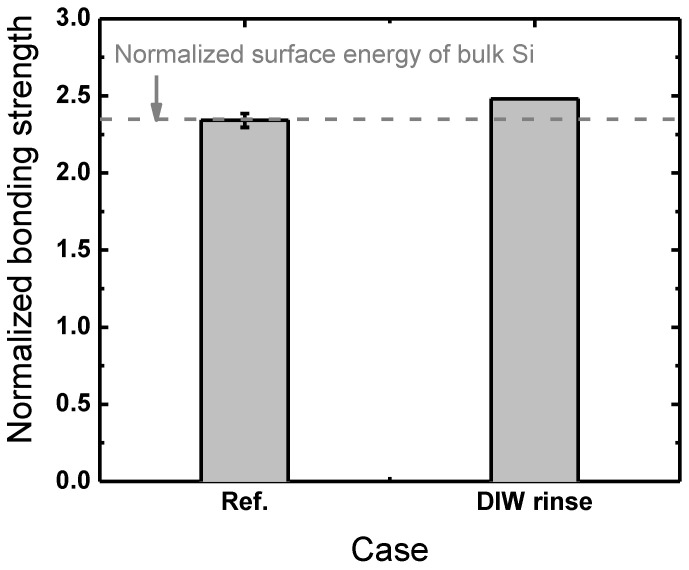
Comparison between the bonding strengths from Si wafers DIW cleaned before and after plasma treatment normalized to the bonding strength with no plasma treatment.

Si-SiO_2_ wafer bonding is often referred to as a hydrophilic bonding due to its fundamental bonding mechanism [33]; when Si and SiO_2_ surfaces are placed together and then annealed, hydroxyl groups (–OH) on each surface are covalently bonded in a form technically termed siloxane bonds (Si-O-Si) [14]. This leads to one idea that a DIW rinsing of the wafer surfaces prior to plasma treatment might benefit the bonding by producing much denser hydroxyl groups on the plasma-treated wafer surfaces, since adsorbed H_2_O molecules could act as a source of hydroxyl groups. DIW rinsing here is conducted in the same manner as the DIW cleaning described above, and we note that only Si surfaces are rinsed while SiO_2_ surfaces are left as-received since a greater DIW rinsing effect is expected on Si over SiO_2_ surfaces due to the higher Si surface hydrophilicity [15]. Figure 4 shows the results of the investigation into the effects of DIW rinsing Si surfaces prior to plasma treatment. It can readily be seen that there is little difference between the bonding strengths with and without preceding DIW rinsing. Since the preceding DIW rinsing is found not to affect the bonding process as much as expected, the following experiments are conducted without the preceding DIW rinsing.

#### 3.1.2. Moisture in Vacuum

As in the case of the preceding DIW rinsing, gas-phase H_2_O in the chamber is also expected to provide a higher surface OH density. To figure out the effects of the chamber H_2_O density, its changes with respect to evacuation times are first monitored via OES with Ar plasma under conditions of 70 sccm flow rate, 100 mTorr pressure, and 100 W RF power. The OES instrument used in this work provides the spectra of detected light in the wavelength range from 200 to 1200 nm. The wavelength of 309 nm has been reported to be emitted from the de-excitation of excited OH molecules, which are chosen in this work as an indicator for the amount of H_2_O molecules [34,35].

**Figure 5:**
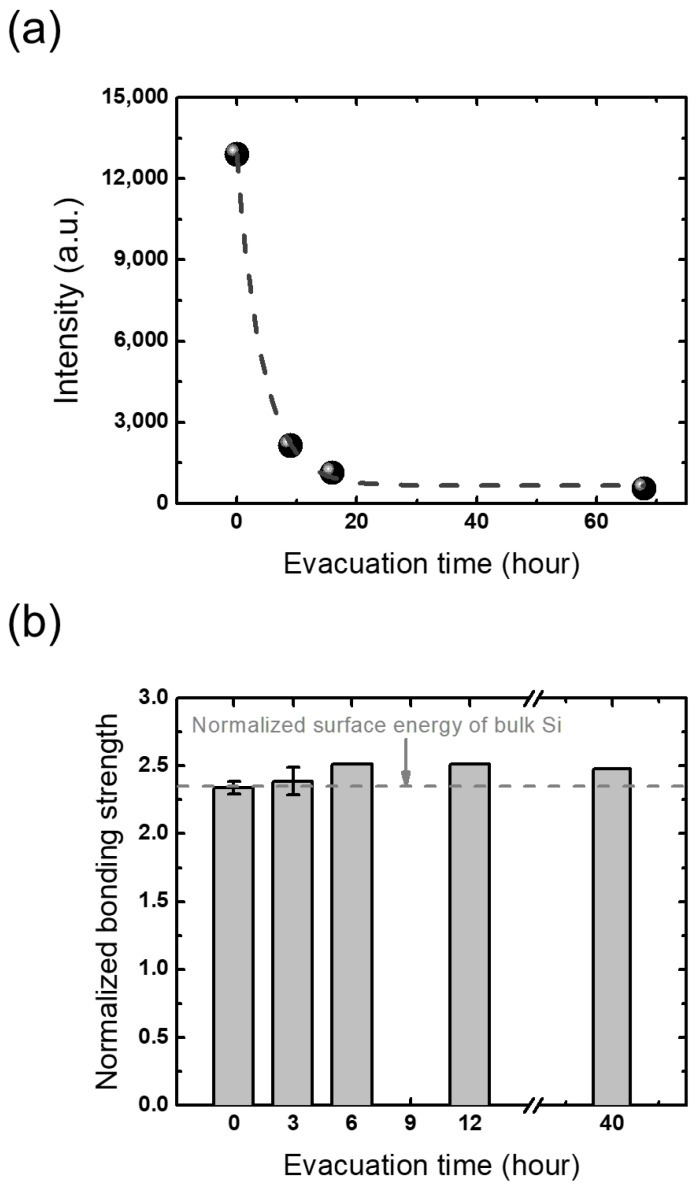
(**a**) Emission intensity of 309 nm wavelength detected by OES as a function of the evacuation time, and (**b**) bonding strengths as a function of the evacuation time normalized to the bonding strength with no plasma treatment.

As plotted in Figure 5a, the OES results show that the 309 nm emission intensity exponentially decays as the evacuation proceeds with a decay constant of approximately 4.3 h. This reflects that H_2_O molecules in the chamber are sufficiently evacuated with 4 h pumping. Figure 5b plots the corresponding DCB results that show bonding strengths obtained with different evacuation times. It is seen that the plasma treatments with evacuation times over 4 h, which is considered to be long enough to pump most H_2_O molecules out of the chamber, produce bonding strengths that are in fact not much different from those obtained with evacuation times shorter than 4 h. This implies that the H_2_O existing in the chamber prior to plasma treatment barely affects the wafer bonding process. From this result, the following experiments are conducted with a 5 min evacuation time.

### 3.2. Plasma Treatment Step

#### 3.2.1. Plasma Parameters

**Figure 6:**
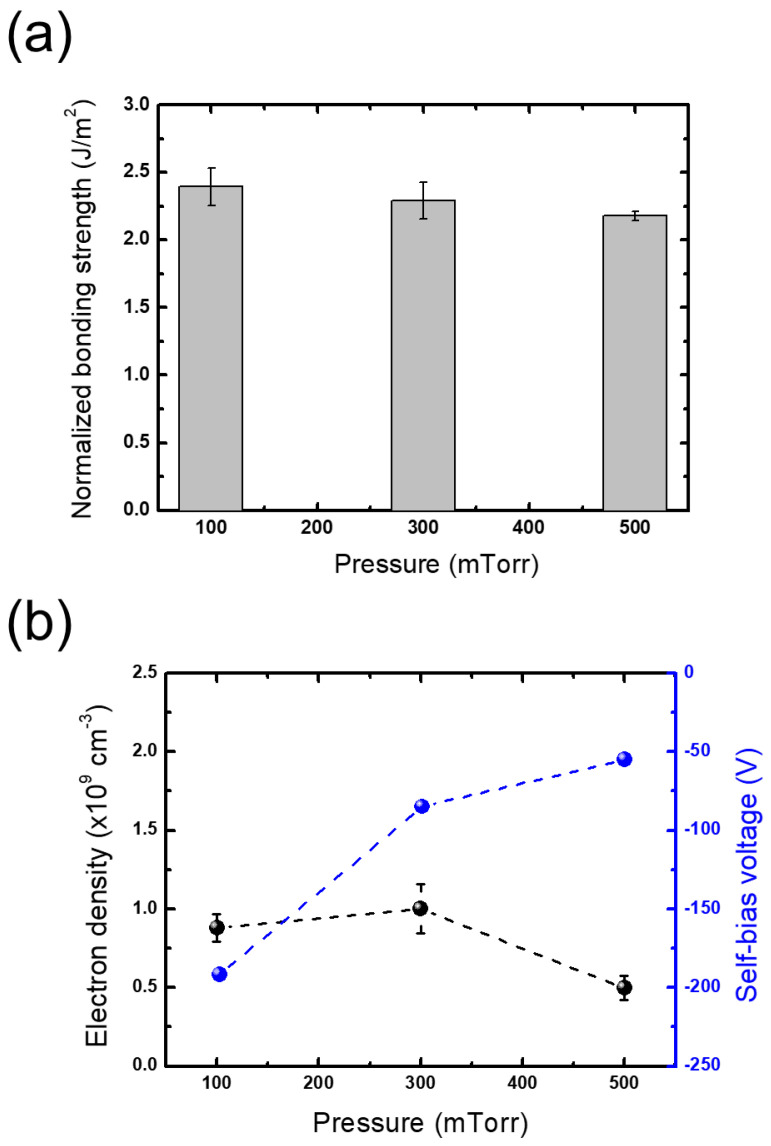
(**a**) Bonding strengths obtained from plasma treatments under different pressures normalized to the bonding strength with no plasma treatment and (**b**) electron density and self-bias voltage measured under different pressures.

Since the effects of plasma treatment can significantly vary according to the properties of the plasma, we investigated changes in bonding strengths obtained under different plasma treatment conditions of pressure and power. Figure 6a shows the changes in bonding strength as the processing pressure increases. For the other conditions except pressure, the reference conditions of the plasma treatment step listed in Table 1 are employed. As shown in Figure 5a, an increase in the pressure leads to a slight decrease in the bonding strength, yet the changes are considered to be negligible, similar to those obtained with different evacuation times.

To interpret this behavior, plasma diagnostics on changes in the electron density and self-bias voltage are conducted. Figure 6b plots the plasma diagnostic results, showing that both the measured electron densities and self-bias voltages slightly decrease with pressure, which implies a decrease in the ion energy flux bombarding the treated wafers. Plach et al. reported that the key role of plasma treatment in wafer bonding is in activating spaces beneath the bonded interface since these spaces act as a water source during bonding, which is advantageous to the formation of siloxane groups in the interface [14]. Thus, the decrease in bonding strength with increasing pressure may be attributed to the decrease in ion energy flux that has a reduced effect on the subsurface regions.

**Figure 7:**
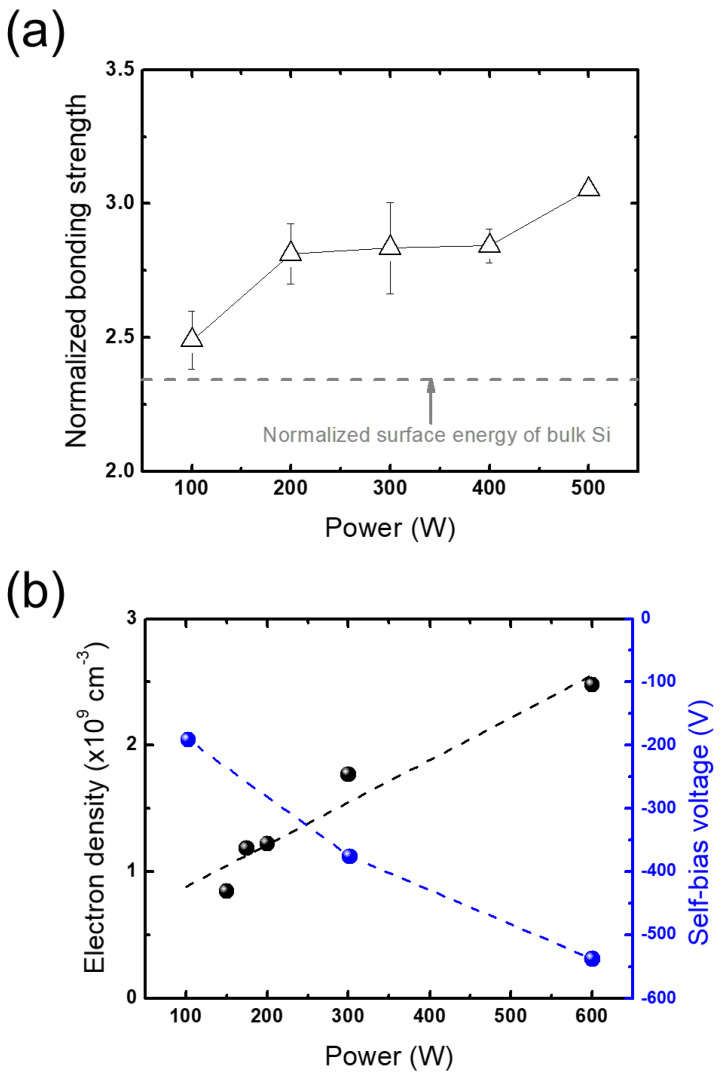
(**a**) Bonding strengths obtained from plasma treatment at different RF powers normalized to the bonding strength with no plasma treatment and (**b**) electron density and self-bias voltage measured at different RF powers.

The above explanation is also applied to the changes in bonding strength with RF power variation. Figure 7a shows the changes in bonding strength with an increase in RF power. Unlike the case of the pressure variation, the bonding strength increases as the RF power increases. This can be elucidated with the plasma diagnostic results shown in Figure 7b; the electron densities and self-bias potentials increase with increasing RF power, reflecting an increase in the ion energy flux. Thus, the increase in bonding strength with increasing RF power can be attributed to the increase in ion energy flux that has a greater effect on the subsurface regions, opposite to the trend with increasing pressure.

**Figure 8:**
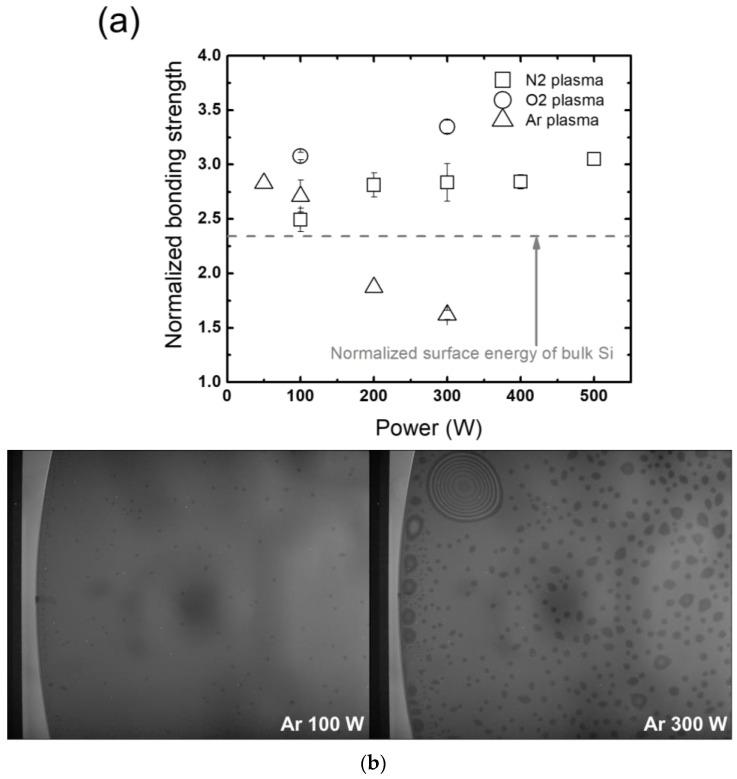
(**a**) Bonding strengths of wafer pairs under different plasma treatments with N_2_, O_2_, and Ar gases, and (**b**) IR images of the bonded interfaces under different plasma treatments with N_2_, O_2_, and Ar gases pictured before and after annealing.

On the other hand, an unexpected trend is observed from employing Ar instead of N_2_ or O_2_ plasma. Figure 8a plots the bonding strength as a function of RF power with Ar and N_2_ plasmas. Note that the data with N_2_ plasma previously shown in Figure 7a are plotted in Figure 8a for ease of comparison. In the figure, it can be seen that when the wafers are treated with Ar plasma, the bonding strength actually decreases with increasing RF power, while O_2_ plasma treatment shows a similar trend as that of N_2_ plasma with slightly increased bonding strengths compared to those obtained with N_2_. Since N_2_ plasma treatment has a wide process window where the bonding strength barely changes with RF power, the assessments in this work are conducted with N_2_. Figure 8b shows IR images of bonded wafers before and after annealing with different gases of N_2_, O_2_, and Ar from different RF powers of 100 W and 300 W for plasma treatment at the 12 o’clock location. The IR images exhibit that Ar plasma treatment with increasing RF power from 100 W to 300 W creates significant annealing voids at the bonded interfaces, while no noticeable changes are detected with N_2_ and O_2_ plasma treatment (not shown). The large extent of the voids created by high-power Ar plasma treatment is attributed to decreasing bonding strength with increasing RF power. This may result from different energy transfer methods, where molecular ions in vibrational states release their energy to the wafer surface while monoatomic ions only transfer their energy via momentum transfer collisions with the surface. Such analysis, though, requires a rigorous investigation for verification.

#### 3.2.2. Wafer-Selective Treatment

**Figure 9:**
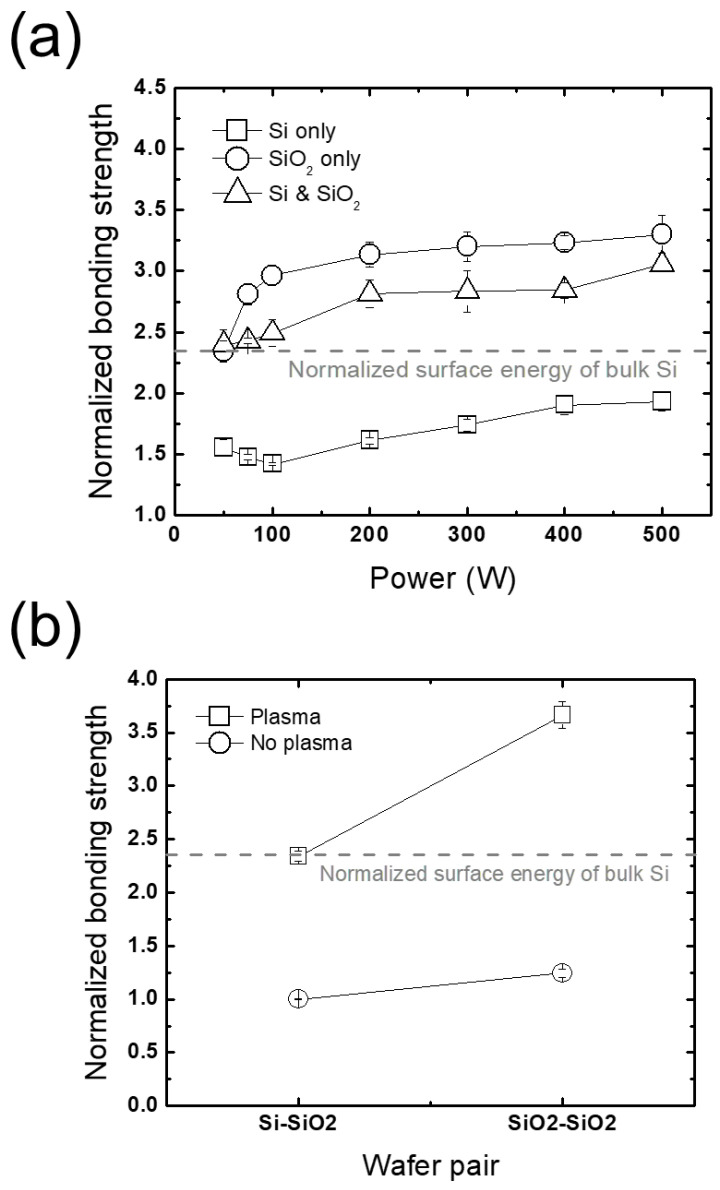
(**a**) Bonding strengths obtained from different plasma surface treatment approaches of Si only, SiO_2_ only, and Si & SiO_2_, and (**b**) comparison between bonding strengths of Si-SiO_2_ and SiO_2_-SiO_2_ pairs with and without plasma treatment normalized to the bonding strength with no plasma treatment.

An investigation into cases where only one side of a Si-SiO_2_ wafer pair is treated with plasma, that is either the Si or the SiO_2_, is conducted to figure out which treatment is more effective. Figure 9a plots a comparison between three cases where both Si and SiO_2_, only Si, or only SiO_2_ wafers are treated (labelled as *Si & SiO_2_*, *Si only*, and *SiO_2_ only* in Figure 9a, respectively) with RF power variation. Note that the *Si & SiO_2_* case is already shown in Figure 6a; it is plotted again here for ease of comparison. With a slight dependence on RF power variation in all cases, the wafer-selective approaches are found to result in remarkable behaviors in terms of the bonding strength; the *Si & SiO_2_* case has lower bonding strengths than the *SiO_2_ only* case, which is an opposite trend to previous reports [14,15]. This disagreement may stem from the difference between the properties of the plasma employed in our work and in the references [14,15]. With a similar self-bias voltage as that in the current work, dual-frequency RF powers in the kHz range were applied to the references’ substrates under a similar pressure. We consider that RF power in the kHz range, which is lower than that used in this work, may produce an ion energy distribution with a wide asymmetric bimodal form where the low-energy population is larger than the high-energy population, resulting in an insufficient ion bombarding energy toward the wafer. Matching the driving frequency in the literature to ours or vice versa is therefore expected to resolve the disagreement.

As shown in Figure 9a, the *Si & SiO_2_* and *SiO_2_ only* cases have increased bonding strengths approximately by a factor of two compared to the Si only case, which means that the plasma treatment on SiO_2_ wafers is much more effective than on Si wafers. The different dependence on plasma treatment between Si and SiO_2_ is also seen in Figure 9b. It is shown that a greater increase in the bonding strength by plasma treatment appears in a SiO_2_-SiO_2_ wafer pair than a Si-Si wafer pair, reflecting the dominant influence of plasma treatment on SiO_2_ over Si in Si-SiO_2_ wafer bonding. This is attributed to the higher porosity of bulk SiO_2_ that enables more water to be stored in the bulk, which strengthens the bonding by inducing a swelling of bulk Si, water stress corrosion, and so on [17,19,36].

A summary of the investigation into the plasma treatment step is as follows. Changes in the plasma parameters with pressure, power, and gas variations are seen to have significant influences on bonding strengths. While an increase in pressure leads to slightly decreased bonding strengths, an increase in RF power gives rise to increased strengths. As for the gas, replacing N_2_ with O_2_ shows a similar trend of increasing bonding strengths with increasing RF power, whereas a noticeable change in the dependence of bonding strengths on RF power is observed with Ar, where the bonding strengths rapidly decrease with increasing RF power.

Single-wafer plasma treatment approaches show that plasma treatment is more effective on SiO_2_ than Si surfaces. Notably, Si treatment in fact degrades the bonding strength, which is an opposite behavior to previous reports [14,15]. As discussed above, this disagreement is considered to stem from the difference between the energy of the plasma ions bombarding the wafer surfaces of our work and the previous reports.

### 3.3. Between Plasma Treatment and Prebonding

Besides the plasma treatment step of the wafer bonding process, factors between the plasma treatment and prebonding steps are also investigated. One of them is a process pause after plasma treatment, which is related to the improved wafer surface hydrophilicity by the plasma treatment. The process pause is investigated following a report that the benefits of plasma treatment in wafer bonding are based on an improvement in hydrophilicity, which reflects a higher silanol group density on wafer surfaces [15]. To evaluate this theory, the wafer bonding process is intentionally paused after the plasma treatment step, with the plasma-treated Si and SiO_2_ wafers temporarily stored in a clean room at ambient atmosphere for 5 days.

**Figure 10:**
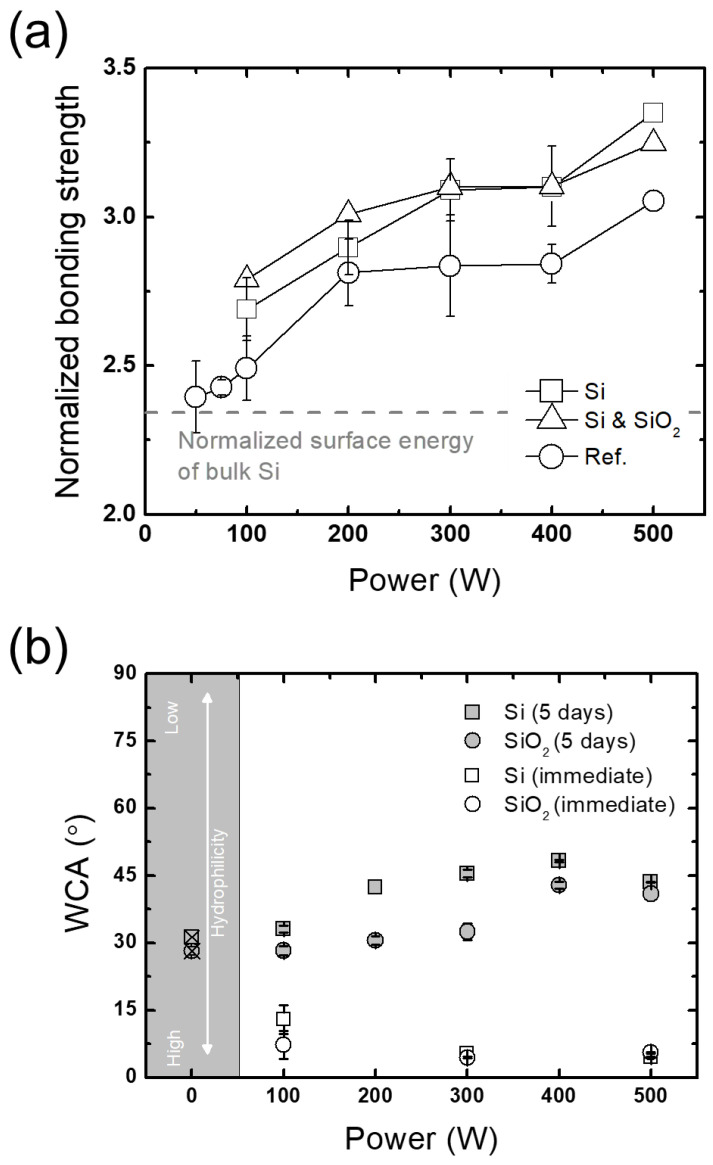
(**a**) Comparison of bonding strengths obtained from wafers stored between plasma treatment and DIW cleaning steps normalized to the bonding strength with no plasma treatment and (**b**) Comparison of water contact angles (WCAs) obtained from plasma treatments at different RF powers.

A comparison between no pause and 5-day pause cases is plotted in Figure 10a. The results show that the bonding strengths of the plasma-treated wafers following a 5-day pause are in fact higher than those with no pause. Water contact angle (WCA) measurements on the wafer surfaces right after and 5 days after plasma treatment are shown in Figure 10b. The results show that the WCAs on the wafer surfaces 5 days after plasma treatment are at a similar level as the original ones measured before plasma treatment, while the WCAs right after plasma treatment are nearly zero. These results reflect that the surface hydrophilicity improved by plasma treatment mostly deteriorates back to the original level.

**Figure 11:**
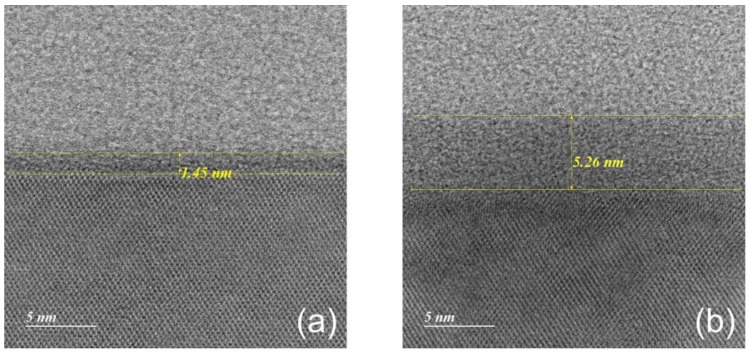
STEM images of the oxide layer on Si measured (**a**) as-received and (**b**) a few days after plasma treatment.

The increase in the bonding strengths obtained by a processing pause of 5 days may be elucidated with the thickness of an oxide layer on Si. Figure 11 shows that plasma treatment with some storage time, sufficiently long for a saturated native oxide to form [37], leads to an increase in the oxide thickness on Si from 1.45 mm to 5.26 nm, while the thermal oxide thickness barely changes (not shown here). Since oxide layers are known to be more porous than Si, the growth of the new oxide layer induced by plasma treatment might assist the following bonding process, leading to the increase in the bonding strengths as plotted in Figure 10a.

### 3.4. Between Prebonding and Annealing

**Figure 12:**
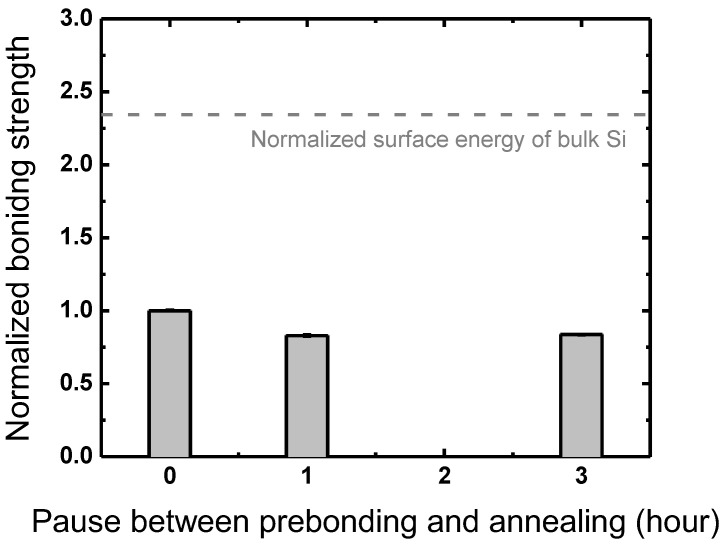
Bonding strengths obtained with different pause durations between prebonding and annealing normalized to the bonding strength with no plasma treatment.

Another process pause is made between the prebonding and annealing steps to figure out the effects of a thin water layer at the interface between prebonded wafers after DIW cleaning. One previous study investigated water diffusion through the bonded interface from ambient atmosphere during storage in air [38], where in scanning acoustic microscopic images of an Si–Si interface, water diffusion from the edge to the center was observed at the interface between two Si wafers immersed in DIW. The gap-filling additional water was found to result in an intense increase in water voids after annealing. The time intervals investigated in the current work, however, do not show much difference between bonding strengths obtained with different pause periods of 0, 1, and 3 h, as shown in Figure 12. Furthermore, the reported water void increase is under the detection limit of the IR images of water voids in this work. Despite the previous report on additional water diffusion to the bonded interface, the time interval between prebonding and annealing is evaluated to have little influence on the bonding strength itself.

### 3.5. Annealing

**Figure 13:**
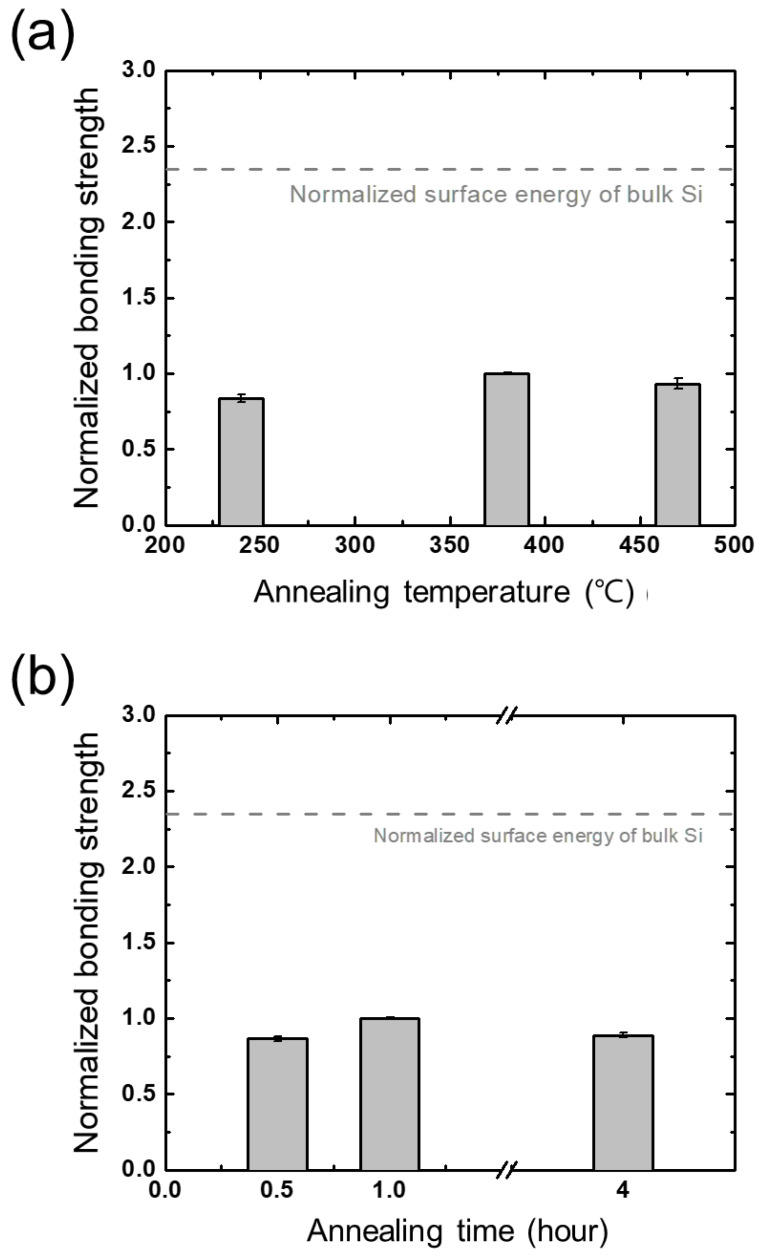
Bonding strengths obtained from different annealing parameters, (**a**) temperature and (**b**) time, normalized to the bonding strength with no plasma treatment.

Annealing is known as one of the most essential process steps in wafer bonding [14,25,33]. The early stages of Si-SiO_2_ bonding employed annealing at high temperatures over 800 °C to obtain a sufficient bonding strength [33]. Such a high-temperature annealing process, however, induced thermal damage in the wafers, which made it difficult for the early direct wafer bonding technique to be adopted [14]. This problem has been dealt with plasma treatment, as numerous studies have reported results of plasma-assisted wafer bonding processes at low temperatures (~300 °C) where sufficient bonding strengths are obtained [16,39]. The effects of annealing temperature and time are evaluated, and the results are shown in Figure 13a,b. Note that these evaluations are conducted with bare Si and SiO_2_ wafers, not plasma-treated. It can readily be seen that the annealing time and temperature do not significantly affect the bonding process, at least within the evaluated regimes.

**Figure 14:**
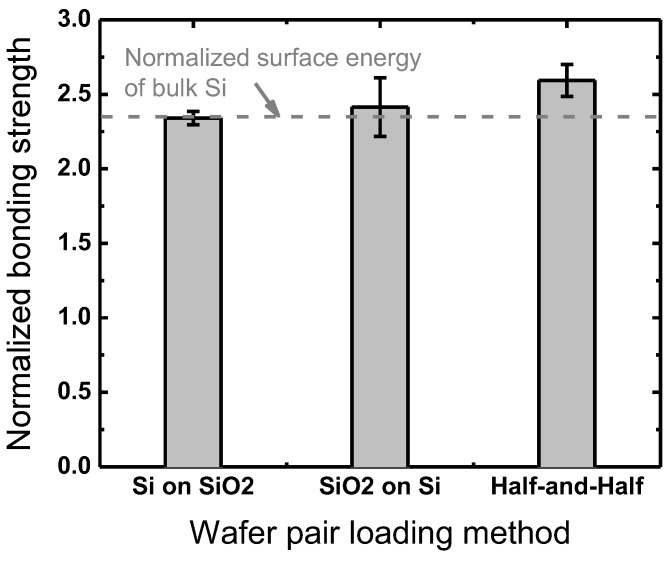
Bonding strengths obtained from different wafer pair loading methods normalized to the bonding strength with no plasma treatment.

Furthermore, wafer pair loading methods into the RTA are evaluated as another factor in the annealing step. While the reference method in this work is a SiO_2_(top)-Si(bottom) stack, the flipped one, Si(top)-SiO_2_(bottom), and a mixed way where the reference stack for half of the annealing period is flipped for the rest of the period are evaluated with plasma-treated Si and SiO_2_ wafers under the reference condition. As plotted in Figure 14, the mixed way provides the highest bonding strength among the three. Considering the annealing method where heat is transferred from the halogen lamps at the top of the RTA to the wafer pair underneath a thick quartz plate, it is reasonable that the top wafer of the pair closer to the quartz plate receives more heat than the bottom wafer, and thus the half-and-half annealing method by flipping the wafer pair during a temporary pause in the middle of annealing results in higher bonding strengths, likely due to more effective thermal activation from the equal provision of heat to both wafers.

## 4. Conclusions

Despite the importance of the role of plasma treatment in wafer bonding processes, studies on the plasma itself in plasma-assisted wafer bonding processes, to our knowledge, still lack basic experimental data such as the effects of the variation of plasma condition parameters on bonding strengths. In this work, comprehensive experimental studies on the plasma parameters as well as the plasma environment were demonstrated. The key findings of this study are as follows. (i) While the density of water molecules or hydroxyl groups in the vacuum chamber for plasma treatment barely affects the resulting bonding strength, that in ambient air improves bonding strengths. (ii) Plasma treatment dominantly affects the bonding strengths among the other steps in the wafer bonding process, and in particular, the ion bombardment energy toward the wafers has a significant influence on bonding strengths. (iii) In Si-SiO_2_ bonding, plasma treatment on the Si surface in fact deteriorates the bonding strength. (iv) Plasmas with Ar instead of N_2_ lead to unfavorable trends in bonding strengths with RF power variation, which implies that thorough investigations into the interactions between plasmas and material surfaces are required for a deeper understanding of the plasma treatment mechanism during wafer bonding.

It should be noted that the mechanism behind the effects of plasma treatment requires more rigorous investigations, which are outside of scope of this paper. Despite this, the results of this work are expected to provide useful insights into plasma-assisted wafer bonding technology.

## Figures and Tables

**Table 1:** List of the examined conditions for the reference conditions in the wafer bonding process.

Processing Step	Conditions
Preparation	No surface cleaning 5 min evacuation
Plasma treatment	100 sccm N_2_ flow rate 100 mTorr pressure 100 W RF power 15 s duration
DIW cleaning	500 RPM for cleaning for 30 s 1800 RPM for drying for 60 s
Annealing	10 min ramping up to 380 °C 60 min annealing up to 380 °C

## Data Availability

The data presented in this study are available on request from the corresponding author.
